# A Genome-Wide Identification of Genes Undergoing Recombination and Positive Selection in *Neisseria*

**DOI:** 10.1155/2014/815672

**Published:** 2014-08-10

**Authors:** Dong Yu, Yuan Jin, Zhiqiu Yin, Hongguang Ren, Wei Zhou, Long Liang, Junjie Yue

**Affiliations:** Beijing Institute of Biotechnology, Beijing 100071, China

## Abstract

Currently, there is particular interest in the molecular mechanisms of adaptive evolution in bacteria. *Neisseria* is a genus of gram negative bacteria, and there has recently been considerable focus on its two human pathogenic species *N. meningitidis* and *N. gonorrhoeae*. Until now, no genome-wide studies have attempted to scan for the genes related to adaptive evolution. For this reason, we selected 18 *Neisseria* genomes (14 *N. meningitidis*, 3 *N. gonorrhoeae* and 1 commensal *N. lactamics*) to conduct a comparative genome analysis to obtain a comprehensive understanding of the roles of natural selection and homologous recombination throughout the history of adaptive evolution. Among the 1012 core orthologous genes, we identified 635 genes with recombination signals and 10 genes that showed significant evidence of positive selection. Further functional analyses revealed that no functional bias was found in the recombined genes. Positively selected genes are prone to DNA processing and iron uptake, which are essential for the fundamental life cycle. Overall, the results indicate that both recombination and positive selection play crucial roles in the adaptive evolution of *Neisseria* genomes. The positively selected genes and the corresponding amino acid sites provide us with valuable targets for further research into the detailed mechanisms of adaptive evolution in *Neisseria*.

## 1. Introduction

Homologous recombination and positive selection are two indispensable sources of genetic variation and play central roles in the adaptive evolution of many bacteria species [1, 2]. Of the two mechanisms, homologous recombination occurs frequently in some bacteria, such as* Streptomyces* [3],* Helicobacter pylori* [4], and* Neisseria* [5], and could possibly speed adaptation by reducing competition between beneficial mutations [6]. There is also evidence for positive selection in specific genes in certain pathogens, such as* Listeria monocytogenes* [7],* Salmonella* [8],* Streptococcus* [9],* Campylobacter* [10], and* Actinobacilus pleuropneumoniae* [11]. These positively selected genes are usually involved in the dynamic interaction between host and pathogen [12, 13].

At present, there are well-developed methods for detecting genes undergoing recombination and selection. Phi [14] and GENECONV [15] are two common methods used to detect recombination based on different statistical tests. The d_N_/d_S_-based method is typically used to estimate the ratio of the rate of nonsynonymous nucleotide substitutions to that of synonymous substitutions [16, 17]. This ratio indicates whether a gene has been under positive selection (**ω** > 1), neutral selection (**ω** = 1), or purifying selection (**ω** < 1). Combined with the codon models developed by Nielsen and Yang [16, 18], which allow variation in **ω** among sites, this method can identify positive selection signals when there are only few positive sites. All these methods will be employed in this study to detect the genes with the history of recombination or positive selection.

*Neisseria* is a genus of bacteria that colonizes the mucosal surfaces of many animals. Of the known 14 species, only 2 species,* Neisseria meningitides* and* Neisseria gonorrhoeae*, are human pathogens; and the remainders are all commensal or nonpathogenic. Until now, there have been many comparative genomic studies on the genomic evolution of these two pathogenic species [5, 19–27]. Homologous recombination has been found to play a key role in the adaptive evolution of* Neisseria*; however, few studies have characterised the effect of positive selection on the* Neisseria* genome. Only two genes, porB [28] and pilE [29], have received attention, and both have undergone strong positive selection pressure. In this study, we used the genome sequences available for the strains of* N. meningitidis, N. gonorrhoeae*, and nonpathogenic* N. lactamica* to investigate the contributions of recombination and positive selection to the evolution of* Neisseria* genomes. Considering the high sequence diversity and open pan-genome, we focused on the core genome genes during our scan for recombined genes and positively selected genes. Statistical tests and a literature review were conducted to determine the association between genes and the properties of this genus.

## 2. Materials and Methods

### 2.1. Data Preparation

Eighteen genome sequences of* Neisseria*, including complete proteomes and the corresponding coding genes, were retrieved from the NCBI Genome database (http://www.ncbi.nlm.nih.gov/genome/bacteria/). Detailed information, such as Genbank ID and genome size, is listed in Table 1. The COGs (clusters of orthologous groups of proteins) functional classification for each proteome was conducted with ID mapping from the Uniprot database [30]. Then, using* Neisseria gonorrhoeae* FA 1090 as the reference genome, stand-alone BLAST was performed against the proteomes of the remaining 17 strains for homologs (sequence identity > 80% and alignment coverage > 80%) of each of the FA_1090 proteins. For each of the core genes from FA_1090, BLAST was performed against all 18 genomes (including the reference genome) with the same thresholds, and multiple copies in any genome were reported and removed from further analysis. The remaining core proteins were defined as the core orthologs of* Neisseria*.

### 2.2. Alignment and Calculation of Nucleotide Diversity, Informative Sites, Codon Bias, d_N_, and d_S_

The orthologous protein sequences were aligned using the method implemented in muscle [31]. Then, multiple codon alignments of genes corresponding to protein sequence alignments were obtained using PAL2NAL [32]. Using the resulting gene alignments, the gene-by-gene number of informative sites and the nucleotide diversity were obtained from the output of the PhiPack program [14].

In this study, the effective number of codons (N_c_) was used to measure the codon bias. The N_c_ value ranges from 20 for the strongest bias to 61 for no bias [33], and the program CodonW (http://sourceforge.net/projects/codonw/) was used to calculate the values of N_c_ for each gene. The number of synonymous nucleotide substitutions per synonymous site (d_S_) and the number of nonsynonymous nucleotide substitutions per nonsynonymous site (d_N_) were estimated from the gene alignments using the program SNAP [34].

### 2.3. Detection of Recombination

Four statistical procedures GENECONV [15], pairwise homoplasy index (Phi) [14], maximum *χ* ^2^ [35], and neighbor similarity score (NSS) [36] were run on the aligned genes to discover the homologous recombination signals. For the analyses of GENECONV, the parameter *g*-scale was set to 1, which allows mismatches within a recombining fragment. The *P* values were calculated from 10000 random permutations of the data. The remaining three programs were implemented in the PhiPack package and were run with default parameters.

### 2.4. Detection of Selection

FastTree [37] was used to construct maximum likelihood phylogenetic trees with a general time-reversible (GTR) model of nucleotide substitution for each gene alignment. The resulting topologies of ML trees were applied to subsequent selection analysis.

The codeml program from PAML [38] was used to detect the genes under positive selection. Two site-specific models were applied: the null model M1a (nearly neutral) and the alternative model M2a (positive selection); the two models differ by the statistical distribution assumed for the **ω** ratio. The latter model allows sites with **ω** > 1, whereas the former only allows sites with **ω** varying between 0 and 1. To ensure convergence to the best likelihood, all calculations were performed three times. A likelihood ratio test (LRT) was then carried out to infer the occurrence of sites under positive selection pressure through comparing M1a against M2a. *P* values were determined from the LRT scores calculated by the module *χ* ^2^ of the PAML package.

### 2.5. Statistical Analysis

Correction for multiple testing was performed using the method presented by Benjamini and Hochberg [39]. For all genes tested for recombination and positive selection, *q*-values were calculated for each *P* value using the *R* package [40, 41] (*q*-value with the proportion of true null hypothesis set to 1). According to the conservation of tests, false discovery rates of 10% and 20% were used for the recombination analyses and positive selection detection, respectively.

The significance level for differences among the properties, including nucleotide diversity, codon bias, d_S_, and d_N_, between a COG and other COGs was determined using the nonparametric Mann-Whitney *U*-test. Correlation between each COG and evolutionary forces (homologous recombination and positive selection) was estimated using a binomial test. Then, Bonferroni corrections for multiple comparisons were performed according to the number of one-sided tests. The significance level was set to 5%. All statistical tests were carried out using Python scripts and *R*.

## 3. Results and Discussion

### 3.1. Characterization of the Orthologous Genes in 18* Neisseria* Genomes

Previous studies [42–45] showed that both intraspecies and interspecies recombination could act as the important genetic mechanism in generating new clones and alleles in* Neisseria*. The genus* Neisseria* consists of two important pathogenic species and a dozen species that are never or rarely pathogenic. At present, there are only 18 completely sequenced genomes of genus* Neisseria* available, including 14* N. meningitidis*, 3* N. gonorrhoeae*, and 1* N. lactamics* genomes. Thus, we selected all 18 genomes to conduct a genome-wide scan for the identification of genes exhibiting recombination or positively selected signals.

**Figure 1:**
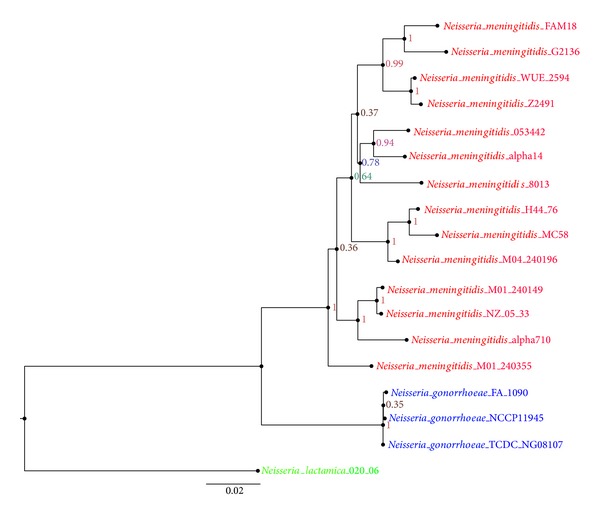
Phylogram of concatenated sequences of 7 housekeeping genes (abcZ, adk, aroE, fumC, gdh, pdhC, and pgm) for the 18* Neisseria* genomes analyzed. The genomes in different species are marked with different colors: red for* Neisseria meningitides*, blue for* Neisseria gonorrhoeae*, and green for* Neisseria lactamics*. The numbers labeled on each internal node are the boostrap values.

The phylogenetic relationships of the 18 strains were first established based on the 7 housekeeping genes frequently used for multilocus sequence typing (MLST) analysis of* Neisseria*: abcZ, adk, aroE, fumC, gdh, pdhC, and pgm [46]. The 7 genes were concatenated to construct a maximum likelihood tree with high bootstrap values as shown in Figure 1. In the tree, the three species were divided into three clades and formed a monophyly, respectively.

In the next step,* N. gonorrhoeae* FA 1090 was used as the reference genome to perform a BLAST search against the other 17* Neisseria* genomes for orthologs. Finally, 1034 genes were identified as present in all 18 genomes, containing the initial definition of the core genome for these* Neisseria* strains and accounting for 38.73% to 55.45% of the coding genes in each genome. This proportion is similar to that in previous analysis of* Neisseria meningitides* genomes [5, 27]. Of the 1034 core genes, 22 genes occurred as two or more copies in some genomes and were excluded from further analysis. The remaining 1012 genes with a single copy per genome were then defined as the core orthologous genes for subsequent analysis of homologous recombination and natural selection.

Among these genes, genes in COGs “Replication, recombination, and repair” were found to show higher nucleotide diversity than genes in other COGs (Table 2). For the association between the number of informative sites and COGs, the same result was obtained, which means genes in category “Replication, recombination, and repair” also had more informative sites than genes in other COGs (Table 2).

The effective number of codons, abbreviated as N_c_, was used to measure the codon bias for each orthologous gene. Genes categorised into the COG “Translation, ribosomal structure and biogenesis” were evident to have a significant higher codon bias compared with genes in other COG categories (Table 2). It is well known that genes with a lower N_c_ can have a strong bias and are more likely to be highly expressed [47–49]. So, the genes in the two COGs might present housekeeping features in the fundamental life cycle and essential physiological activities of* Neisseria*.

In the same way, an association between COGs and d_N_ or d_S_ was also observed. There were 4 COGs in which genes were found to have higher rates of synonymous nucleotide substitutions in comparison with other categories. On the other hand, genes in the other 4 COGs also showed a tendency to have higher rates of nonsynonymous substitutions in comparison with genes in other COGs (Table 2). It is worth noting that all the genes in the core genome in* Neisseria* had higher d_S_ and d_N_ rates than the genes in other bacteria, for example,* E. coli* [50] and* A. pleuropneumoniae* [11], indicating that strong natural selection might act on* Neisseria*.

### 3.2. A Considerable Number of Genes Showing Evidence of Recombination

Until now, there were several different strategies for identifying the homologous recombination regions in sequences. In this study, four common statistical test methods, including NSS, Max-*χ*2, Phi, and GENECONV, were employed to detect the recombination signals among the 1012 orthologous genes. As a result, a total of 996 genes (98.4% of all 1012 core genome genes) were found to show significant evidence (FDR < 10%) of recombination by at least one of the four tests. Overall, 951, 968, 842, and 727 genes were identified to show significant evidence of recombination by NSS, Max-*χ*2, Phi, and GENECONV, respectively. Additionally, a total of 635 genes (62.7% of 1012 core genome genes) were showed recombination signals in all four tests. The proportion of genes undergoing recombination ranged from 62.7% to 98.4%, which is higher than those typically observed in other bacteria, such as* E. coli*. The result suggests that homologous recombination plays an important role in the evolution of* Neisseria* genomes.

In a previous work [5], Joseph et al. identified 459 ortholog genes with signs of recombination in* Neisseria meningitidis* genomes, which accounts for 39.6% of all core genome genes. In this work, only* Neisseria meningitidis* genomes were for recombination test, the abovementioned 459 orthologous genes with signs of recombination could be considered intraspecies recombinations. In our present work, in addition to the* N. meningitidis* genomes, the genomes of* Neisseria gonorrhoeae*, and* Neisseria lactamica* were also selected for the recombination analyses and several interspecies recombination genes were identified. The interspecies recombination events in the genus* Neisseria* have been reported many times [44–46]. It is not surprising that the proportion of genes with recombination signals in the present work is markedly higher than the value observed by Joseph et al. It can be deduced that both intraspecies and interspecies recombination could act as important genetic mechanisms for generating new clones and alleles [47] in* Neisseria*.

To test whether the high percentage of core genome genes with a recombination signal is caused by the choice of genomes, we carried out the same analysis on the 14* N. meningitidis* genome sequences with the same parameters. We first obtained 1211 orthologous genes with a single copy per genome. Among these orthologous genes, 634 (52.4%) genes were identified to show significant evidence of recombination by all the four tests. In this case, a lower percentage of genes with recombination signals were identified, confirming that the choice of genomes really has an impact on the percentage of recombined genes in the core genome. It also indicated that interspecies recombination indeed has a role in the evolution of* Neisseria* genomes. Additionally, a higher proportion of genes with recombination signals were observed in these 14* N. meningitidis* genomes compared with the results in Joseph's work. The reason could lie in the differences in the specific genomes in both analyses, suggesting that intraspecies recombination plays an unexpected role in the evolution of the* N. meningitidis* genome. In a word, recombination acts as an important and irreplaceable genetic mechanism in shaping the genomes of genus* Neisseria*.

**Figure 2:**
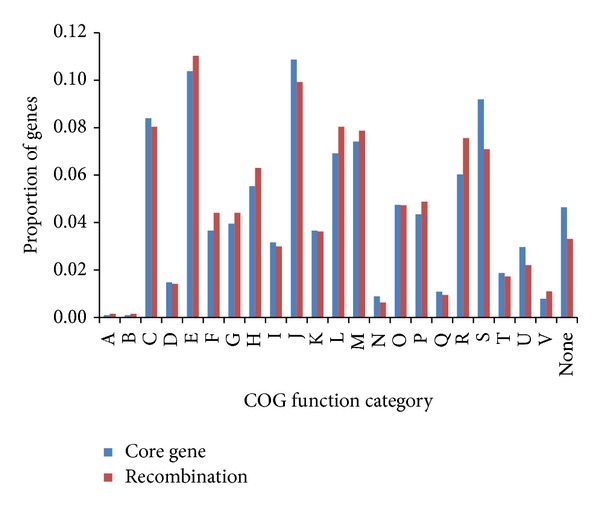
Genes with recombination signals are distributed with no significance in all COGs. The *x* axis represents different COG categories. The *y* axis represents the proportion of genes in each COG category. The proportion of genes with evidence for recombination and core genes for each COG are represented by red and blue bars, respectively. The COG categories are coded as follows: A, RNA processing and modification; B, chromatin structure and dynamics; C, energy production and conversion; D, cell cycle control, cell division, and chromosome partitioning; E, amino acid transport and metabolism; F, nucleotide transport and metabolism; G, carbohydrate transport and metabolism; H, coenzyme transport and metabolism; I, lipid transport and metabolism; J, translation, ribosomal structure and biogenesis; K, transcription; L, replication, recombination, and repair; M, cell wall/membrane/envelope biogenesis; N, cell motility; O, posttranslational modification, protein turnover, and chaperone; P, inorganic ion transport and metabolism; Q, secondary metabolites biosynthesis, transport, and catabolism; R, general function prediction only; S, function unknown; T, signal transduction mechanisms; U, intracellular trafficking, secretion, and vesicular transport; V, defense mechanisms; None, not in COGs.

Moreover, it is worth noting that the core genes identified as recombinants have high rates of d_S_ and d_N_, nucleotide diversity and the number of information sites (*P* < 0.001, *P* < 0.001, *P* < 0.001 and *P* < 0.001, respectively, one-sided *U*-test). The association between COG categories and the number of recombined genes was also estimated (Figure 2). Only two COGs “general function prediction only” and “function unknown” were significantly overrepresented with recombined genes. However, after Bonferroni correction, all the genes exhibiting evidence of recombination were distributed with no significance in all COGs. This unbiasedness of recombined genes in function further confirmed the role of recombination in shaping genomes during the evolution of* Neisseria*.

### 3.3. 10 Genes Showing Evidence of Positive Selection

The detection of positive selection for the 1012 orthologs was conducted in PAML, and models M1a and M2a of variable selective pressure across codon sites were used to estimate selective pressure and test for positive selection. Based on LRT statistics for comparing the null model and alternative model with *χ* ^2^ distribution and correction for multiple testing (FDR < 20%), a total of 10 genes were identified to be under strong selected pressure. Of the 10 genes, 4 belonged to the COG “Replication, recombination, and repair”, and 3 were in the COG “Inorganic ion transport and metabolism.” The remaining three genes were classified into the “cell wall/membrane/envelope biogenesis,” “nucleotide transport and metabolism,” and “function unknown”, respectively (Table 3).

In the same way, two obvious discrepancies were observed, respectively, for values of d_S_ and the number of informative sites between genes under positive selection and the remaining genes (*P* = 0.024 and *P* = 0.005, one-sided *U*-test). Furthermore, all 10 positively selected genes were found to show significant evidence of recombination detected by at least one recombination test. Only one gene was not in the genes identified by all four tests. The probable reason for this is that recombination could form phylogenetic incongruence [51, 52].

Compared to the high proportion of recombined genes, few positively selected genes (10) were identified, accounting for approximately 1% of the core genome. Similar proportion was also obtained in* E. coli* [12], but is smaller than those of other pathogenic bacteria, such as* A. pleuropneumoniae* [11].

Among the protein products encoded by the 10 positively selected genes, only 8 proteins were annotated with definite functions. We found that these proteins were either involved in DNA processing or inorganic transport and metabolism.

Of the 10 genes, recB, encoding the DNA helicase, is an integral part of recBCD homologous recombined enzyme. Mutations in recB are required for double-strand break repair [53] and can also reduce the frequency of many types of recombination events [54]. dnaE, dnaX and polA are all DNA polymerase genes. The first two encode the polymerase iii subunits, and the last encodes polymerase I. All three play fundamental roles in DNA metabolism, including DNA replication, recombination, and repair. In a word, positive selection on the four genes might ensure the strain to adapt to frequent recombination in the genomes.

AmtB encodes an ammonium transporter and is involved in ammonium transmembrane transporter activity. uraA encodes a uracil permease involved in transmembrane transport as well and acts as a membrane-bound facilitator for the transport of uracil across the cell membrane into the cytoplasm [55]; it is therefore necessary for uracil uptake, especially at low exogenous uracil concentrations and even under conditions with high UPRTase activity.

Hup encodes a TonB-dependent receptor that utilizes heme as an iron source [56]. It has been reported that mutations in the hemoglobin receptor gene have profound effects on the survival of* N. meningitidis* in an infant rat, indicating that this gene is important for the virulence of* Neisseria* [57].

FrpB is clearly a virulence gene, encoding an iron-regulated outer membrane protein. It is a member of the TonB-dependent transporter family and is responsible for iron uptake into the periplasm. FrpB is subject to a high degree of antigenic variation, principally through a region of hypervariable sequence exposed on the cell surface [58, 59].

In a word, the four genes play important roles in the uptake of nutrition. So the adaptive changes in these proteins might be beneficial for* Neisseria* to survive in the host.

## 4. Conclusion

Our analysis reported here indicates that both homologous recombination and positive selection play important roles in the evolution of the core genome in* Neisseria*. Additionally, homologous recombination has a greater contribution to the genetic variation of a large number of genes with recombination signals. Only 10 genes were identified to be under positive selection, which also showed significant evidence of recombination. However, the positively selected genes were found to be involved in DNA processing or located on the cell membrane. The former reduce the frequency of recombination and enables a stable genetic environment, while the latter maintain a dynamic interaction with the external environment, as well as with the host. Overall, the changes in these positively selected genes result in an improvement in bacterial fitness in response to a variety of environmental signals. These genes can be regarded as a screened gene set for further analysis of the mechanisms of adaptive evolution in* Neisseria*.

## Figures and Tables

**Table 1:** Genome sequences used in this study.

Strain name	GenBank accession no.	Genome size (Mbp)	No. of CDS	CC ID
*Neisseria_meningitidis*_FAM18	NC_008767	2.19	1917	CC11
*Neisseria_meningitidis*_G2136	NC_017513	2.18	1928	CC8
*Neisseria_meningitidis*_WUE_2594	NC_017512	2.23	1941	CC5
*Neisseria_meningitidis*_Z2491	NC_003116	2.18	1909	CC4
*Neisseria_meningitidis*_8013	NC_017501	2.28	1913	CC18
*Neisseria_meningitidis*_053442	NC_010120	2.15	2020	CC4821
*Neisseria_meningitidis*_alpha14	NC_013016	2.14	1872	CC53
*Neisseria_meningitidis*_M04_240196	NC_017515	2.25	1947	CC269
*Neisseria_meningitidis*_H44_76	NC_017516	2.24	1961	CC32
*Neisseria_meningitidis*_MC58	NC_003112	2.27	2063	CC32
*Neisseria_meningitidis*_alpha710	NC_017505	2.24	2017	CC41/44
*Neisseria_meningitidis*_M01_240149	NC_017514	2.22	1936	CC41/44
*Neisseria_meningitidis*_NZ_05_33	NC_017518	2.24	1948	CC41/44
*Neisseria_meningitidis_*M01_240355	NC_017517	2.29	1971	CC213
*Neisseria_gonorrhoeae*_TCDC_NG08107	NC_017511	2.15	2196	
*Neisseria_gonorrhoeae*_FA_1090	NC_002946	2.15	2002	
*Neisseria_gonorrhoeae*_NCCP11945	NC_011035	2.23	2680	
*Neisseria_lactamica*_020_06	NC_014752	2.22	1972	

**Table 2:** Association between COGs and descriptive variables.

Functional category	Number of genes analyzed	Bonferroni-corrected *P* value for one-sided *U*−test for association between genes in a given COG and^(1)^					
		>nt diversity	>Number of Informative sites	>Codon bias^(2)^	<Codon bias^(2)^	>d_S_	>d_N_
Energy production and conversion	85						**<0.001**
Nucleotide metabolism and transport	37					**0.03**	
Translation, ribosomal structure, and biogenesis	110			**<0.001**		**0.03**	
Replication, recombination, and repair	70	**<0.001**	**<0.001**				**0.03**
Cell wall/membrane/envelope biogenesis	75					**0.002**	
Function unknown	93				**0.023 **		**0.03**
Intracellular trafficking, secretion and vesicular transport	29				**0.020 **	**0.039**	
Not in COGs	47				**0.040 **		**<0.001**

^(1)^“>” or “<” indicates the direction of the one-sided tests (i.e. “>Codon bias” shows Bonferroni-corrected *P*-values for associations between genes in a given COG and higher codon bias as compared to the genes in other COGs, and “<Codon bias” represents a contrast tendency).

^(2)^Tests for codon bias were performed using N_c_ values (a lower N_c_ means increased codon bias).

**Table 3:** Genes under positive selection.

Gene	Cluster ID	COG	Function	2Δ*L*	*q*-value	*ω*	Positively selected sites
dnaE	N35	L	DNA polymerase III alpha subunit	48.459	0.016	13.082	413, 968, 971, 972
	N139	P	Ammonium transporter	42.474	0.096	63.631	12, 14, 18, 19, 20, 21, 67
recB	N245	L	DNA helicase	67.811	0.000	4.287	4, 251, 865, 869, 882, 1036, 1137, 1184
hup	N352	P	TonB-dependent receptor	129.195	0.000	7.064	263, 265, 282, 287, 288, 290, 291, 293, 304, 378, 380, 535, 538, 553, 557, 561, 646, 810, 884, 889, 891
	N380	M	Hypothetical protein	46.418	0.029	152.674	18, 19, 20, 23, 24, 25, 26, 28, 30
dnaX	N436	L	DNA polymerase III gamma and tau subunit	57.997	0.001	6.032	228, 294, 329, 512, 559
uraA	N514	F	Uracil permease	55.222	0.002	16.737	2, 9, 10, 17, 24, 25, 29, 31, 395, 455
	N832	S	Hypothetical protein	51.544	0.006	6.580	190, 207, 212, 228, 232, 276, 314, 368, 401, 514, 729
frpB	N966	P	Iron-regulated outer membrane protein	125.098	0.000	4.333	341, 342, 343, 348, 394, 409, 415, 451, 459, 466, 467, 471, 472, 473, 476, 483, 674, 688, 718, 730, 739
polA	N973	L	DNA polymerase I	54.283	0.003	6.489	212, 866, 867, 881, 882, 898
